# Cardiorenal Syndrome: New Pathways and Novel Biomarkers

**DOI:** 10.3390/biom11111581

**Published:** 2021-10-26

**Authors:** Guido Gembillo, Luca Visconti, Maria Ausilia Giusti, Rossella Siligato, Alessia Gallo, Domenico Santoro, Alessandro Mattina

**Affiliations:** 1 Unit of Nephrology and Dialysis, Department of Clinical and Experimental Medicine, University of Messina, 98125 Messina, Italy; rossellasiligato@gmail.com; 2 Department of Biomedical and Dental Sciences and Morpho-Functional Imaging, University of Messina, 98125 Messina, Italy; 3 Unit of Nephrology and Dialysis, Ospedali Riuniti Villa Sofia Cervello, University of Palermo, 90146 Palermo, Italy; lucavisconti2003@yahoo.it; 4 Diabetes and Islet Transplantation Unit, Department of Diagnostic and Therapeutic Services, IRCCS-ISMETT (Istituto Mediterraneo per i Trapianti e Terapie ad alta Specializzazione), UPMC Italy, 90127 Palermo, Italy; giustiausilia@gmail.com (M.A.G.); alessandromattina@gmail.com (A.M.); 5 Department of Research, IRCCS ISMETT (Istituto Mediterraneo per i Trapianti e Terapie ad alta Specializzazione), UPMC Italy, 90127 Palermo, Italy; agallo@ismett.edu

**Keywords:** cardiorenal syndrome, acute kidney injury, chronic renocardiac syndrome, novel biomarkers, heart, kidney, renal injury, NGAL, KIM-1, proBNP, microRNA

## Abstract

Cardiorenal syndrome (CRS) is a multi-organ disease characterized by the complex interaction between heart and kidney during acute or chronic injury. The pathogenesis of CRS involves metabolic, hemodynamic, neurohormonal, and inflammatory mechanisms, and atherosclerotic degeneration. In the process of better understanding the bi-directional pathophysiological aspects of CRS, the need to find precise and easy-to-use markers has also evolved. Based on the new pathophysiological standpoints and an overall vision of the CRS, the literature on renal, cardiac, metabolic, oxidative, and vascular circulating biomarkers was evaluated. Though the effectiveness of different extensively applied biomarkers remains controversial, evidence for several indicators, particularly when combined, has increased in recent years. From new aspects of classic biomarkers to microRNAs, this review aimed at a 360-degree analysis of the pathways that balance the kidney and the heart physiologies. In this delicate system, different markers and their combination can shed light on the diagnosis, risk, and prognosis of CRS.

## 1. Introduction

### Definition of Cardiorenal Syndrome

There is a close link between the heart and kidney: cardiovascular damage drives a worsening of kidney function; in turn kidney failure worsens cardiovascular injury [1]. Heart Failure (HF) affects 5.8 million people within the USA, and over 23 million worldwide [2]. Approximately 20–30% of patients admitted for acute decompensated HF will suffer a decline in kidney function while hospitalized, and 40–60% of patients with chronic HF have CKD. The coexistence of these two diseases worsens their prognoses [3,4].

Cardiorenal syndrome (CRS) is a multi-organ disease that includes a spectrum of disorders resulting from the close interaction between the heart and kidney during acute or chronic dysfunction of one of these organs.

It was described for the first time by Robert Bright, in 1836, who found cardiac structural changes in a patient with advanced renal failure [5]. Since then, several cases have been reported, and the knowledge of its pathophysiology has gradually led to a better clinical classification of the syndrome. In 2004, the Working Group of the National Heart, Lung, and Blood Institute defined, for the first time, CRS as “the result of interactions between the kidneys and other circulatory compartments that increase circulating volume, which exacerbates the symptoms of heart failure and disease progression” [6]. In this context, the heart is considered to be the main actor, and in which the decline of renal function worsens the congestive symptoms of HF.

The bidirectional nature of CRS has recently been highlighted, and the common pathogenesis identified in hemodynamic, neurohormonal, inflammatory mechanisms also involving the atherosclerotic degeneration.

**Figure 1:**
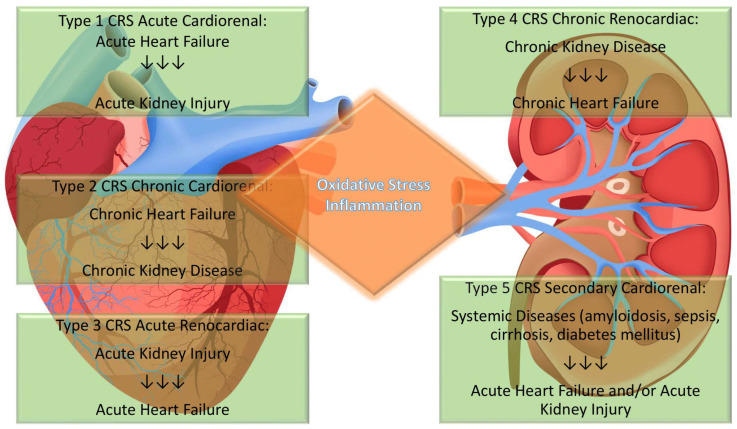
Different Types of Cardiorenal Syndromes. CRS presents five subtypes characterized by the influence of chronic or acute dysfunction of one organ on another. The acute dialysis quality initiative classification of CRS is helpful to drive the clinicians to the best therapeutic strategies depending on the primum movens of the syndrome. The complex interconnection of this condition is characterized by oxidative stress and inflammation-driven damage.

In 2008, the Acute Dialysis Quality Initiative divided CRS patients into two groups based on the primary organ failure driving the disease process (cardiorenal or renocardiac) [7]. This classification was further elaborated and arranged into five groups according to clinical presentations to facilitate the early differential diagnosis and the most appropriate therapy (Figure 1), and biomarkers may represent decisive prognostic factors in assessing risk prediction in patients with HF and impaired kidney function.

## 2. Pathophysiology of Cardiorenal Syndrome

Multiple processes are involved in the pathophysiological mechanism of CRS. Each of these plays a fundamental role in CRS development and cannot be considered an isolated entity but, on the contrary, a portion of larger multifaceted and complex pathophysiological pathways.

### 2.1. Hemodynamic Mechanism

In acute decompensated heart failure, the cardiac pump cannot maintain optimal blood flow, resulting in volume overload and increased central venous pressure (CVP). CVP is the thoracic vena cava’s pressure near the right atrium. It indicates the blood volume returning to the heart and its ability to pump the blood back into the arterial system. When this mechanism fails, consequent venous congestion results in renal dysfunction caused by reduced renal blood flow and glomerular filtration rate (GFR), and decreased urine output.

Damman et al. [8] evaluated a total of 2557 patients who underwent right heart catheterization. They found that CVP was associated with estimated GFR (eGFR) reduction (*p* < 0.0001). In addition, on multivariate analysis, CVP remained associated with eGFR (*p* < 0.0001). Furthermore, they showed that CVP was an independent predictor of reduced survival rate (*p* = 0.0032). A recent meta-analysis included fifteen cohort studies, and showed that elevated CVP was associated with an increased risk of mortality (three studies; 969 participants; OR, 1.65; 95% CI, 1.19–2.29), and acute kidney injury (AKI) (two studies; 689 participants; OR, 2.09; 95% CI, 1.39–3.14) [9].

**Figure 2:**
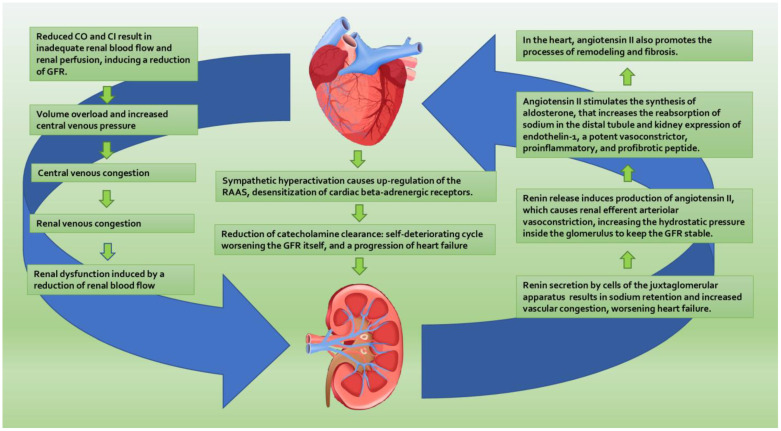
Bidirectional mechanisms in cardiorenal axis pathophysiology. CRS presents a common pathogenesis characterized by hemodynamic, neurohormonal, inflammatory mechanisms also involving the atherosclerotic degeneration. RAAS system plays a major role in CRS pathophysiology, tipping the scale in the delicate balance between heart and kidney. Abbreviations: CI: cardiac index, CO: cardiac output, GFR: glomerular filtration rate, RAAS: renin–angiotensin–aldosterone system.

Two other parameters to consider in the direct hemodynamic mechanisms of CRS are cardiac output (CO) and cardiac index (CI). CO is the volume of blood pumped by the heart per minute. It is the result of the product of the heart rate and stroke volume. The normal range for cardiac output is about 4 to 8 L/min. CI is a hemodynamic parameter that relates cardiac output to the patient’s size. The normal range for CI is 2.5 to 4 L/min/m^2^. A reduced CO and CI result in inadequate renal blood flow and poor renal perfusion that determine, per se, a reduction of GFR. Furthermore, it leads to renin secretion by cells of the juxtaglomerular apparatus, which results in sodium retention and increased vascular congestion, with worsening heart failure (Figure 2). 

A randomized controlled trial (ESCAPE) included 433 patients with heart failure to determine whether pulmonary artery catheter use is safe and improves clinical outcomes. As a secondary analysis, the investigators found no correlation between CI and renal function [10].

On the contrary, Mullens et al. [11] evaluated 145 consecutive patients ≥18 years with advanced decompensated heart failure (ADHF), and compared those who developed AKI versus those who did not. All patients presented signs of impaired hemodynamics, with impaired CI. The study showed a statistically significant correlation between baseline CI and baseline renal function expressed by serum creatinine (*p* = 0.001) or GFR (*p* = 0.002) [11].

A prospective multicenter study, recruiting 154 patients with cardiogenic shock, found that the lowest CI, and other hemodynamic modifications, were associated with a higher incidence of AKI [12].

### 2.2. Current AKI Biomarkers and New Standpoints

Classical biomarkers for the assessment of kidney function include serum creatinine (SCr), albuminuria, and cystatin C (CysC), as well as urine output and eGFR. Urine and serum biomarkers, including urinary angiotensinogen, urinary enzyme N-acetyl-β-d-glucosaminidase (NAG), neutrophil gelatinase-associated lipocalin (NGAL), interleukin 18 (IL-18), high sensitivity troponin I (hs-cTnI), and kidney injury molecule 1 (KIM-1) measured at the time of CRS diagnosis show improved risk stratification in determining which patients will experience adverse outcomes [13]. Among these, NGAL is the most widely used, and its urinary determination has been found to be more sensitive and specific than even plasma NGAL [14]. The increase in NGAL levels has a predictive role for the onset of acute renal injury, considerably anticipating the increase of creatinine serum levels and thus allowing prompt preventive therapeutic strategies [15].

Though the effectiveness of different extensively applied biomarkers remains controversial, evidence for several indicators, especially when combined, has increased in recent years.

An example is represented by the reduced levels of potassium and albuminuria, which have been shown to be related to early renal repair processes after cardiac resynchronization therapy (CRT) in CRS Type 2. In a population of 76 patients (15% female; aged 69 ± 9.56 years) with HF (New York Heart Association classes II-IV), the amount of these two biomarkers was reduced after CRT (*p* < 0.05), while urinary NGAL, eGFR, and SCr were unvaried [16].

Interestingly, Tasić et al. [17] demonstrated that increased xanthine oxidoreductase activity (XOA), elevated CysC values, and GFR were significantly related to risk of acute CRS. They studied a population of 79 patients, dividing them into subgroups depending on their acute or chronic CRS presentation. The results showed a significant risk for acute CRS with increased XOA (OR = 1.015; 95% CI 1.001–1.030, *p* = 0.037), elevated CysC concentration (OR = 1.821; 95% CI 1.034–3.205, *p* = 0.038), and reduced GFR (OR = 0.970; 95% CI 0.943–0.997, *p* = 0.028). Conversely, previously validated markers of stress in diabetic and hypertensive nephropathy, such as advanced oxidation protein products (AOPP) [18] and plasminogen activator inhibitor-1 (PAI-1) [19] showed no significance for the occurrence of either acute or chronic CRS in their population. 

The severity of CRS has been demonstrated to be potentially linked to β2-microglobulin (b2M) and tissue inhibitor of metalloproteinases 1 (TIMP 1) levels. Atici et al. [20] found that b2M level correlated with the severity of cardiorenal impairment and with proBNP (*r* = 0.66, *p* > 0.0007), percent ejection fraction (*r* = −0.56, *p* = 0.0162), and GFR (*r* = 0.83, *p* < 0.0001). Moreover, b2M and TIMP 1 were correlated (*r* = 0.5287, *p* < 0.0095) [21]. Another metallopeptidase inhibitor, TIMP 2, has been tested in association with the insulin-like growth factor-binding protein 7 (IGFBP7) in 111 patients with acute decompensated HF. Patient were divided into two groups according to the occurrence or not of CRS and IGFBP7, TIMP-2, and [TIMP-2]∙[IGFBP7] values were significantly higher in patients with CRS (0.40 (0.25–0.71), p1: 0.049/2.40 (1.42–3.70), p2: 0.003/1.15 (0.29–2.43), p3: 0.001). Kashani et al. [22] conducted a study on a large cohort of 375 heterogeneous, critically ill, postoperative surgical patients, and found that [TIMP-2]∙[IGFBP7] can predict the onset of moderate/severe AKI. Consisting with this evidence, an analysis kit evaluating the combination of urinary TIMP-2 and IGFBP7 levels are currently available.

The urinary level of the liver-type fatty acid-binding protein (L-FABP), a protein expressed by both liver and renal proximal tubule cells, is higher in patients with HF associated with AKI, and is the only one found to be an independent predictor of AKI [23].

The need for an easy-to-perform and effective index obtained with routine blood test, found in the mean platelet volume (MPV) a promising solution in clinical practice. This method has been proposed by Li et al. [24], using MPV as a marker of in-hospital mortality in patients with acute CRS receiving continuous renal replacement therapy. The area under the curve (AUC) value of MPV as a predictor of in-hospital mortality was 0.735 (*p* < 0.05). The rationale for this result can be partially explained by the higher level of prothrombotic cytokines contained in large platelets: these agents will more easily impair endothelial function, with a worsening of inflammation and CV outcomes, as demonstrated in previous studies [25].

### 2.3. Neurohormonal Dysregulation

Both HF and CKD are characterized by abnormal alteration of the sympathetic nervous system (SNS). Sympathetic hyperactivity is usually a compensatory system in the acute phases of CVD that, continuing over time, has cardio and nephrotoxic effects [26]. In CKD, in addition to up-regulation of the renin-angiotensin-aldosterone system (RAAS), sympathetic hyperactivation causes desensitization of cardiac beta-adrenergic receptors; furthermore, catecholamine clearance is reduced, resulting in a self-deteriorating cycle that worsens the GFR itself, and a progression of heart failure [27,28].The direct effects of sympathetic hyperactivation also include the alteration of cardiac calcium homeostasis, increased hypertrophy, and apoptosis of myocytes. This results in the increased predisposition to the chronicization of cardiac remodeling [29]. Indirect effects are essentially mediated by inflammatory molecules, such as cytokines [30].

RAAS activation has important effects in the pathogenesis of CRS. The hypoperfusion of peripheral tissue in HF determines the sympathetic nervous system’s overactivity, with increased renin release from the juxtamedullary apparatus. Renin synthesis is also stimulated by the reduction of hydrostatic pressure at glomerular afferent arterioles, and a reduction of sodium delivered to the macula densa. The result of renin release is the increased production of angiotensin II, which causes renal efferent arteriolar vasoconstriction, increasing the hydrostatic pressure inside the glomerulus to keep the GFR stable. Consequently, it decreases the peritubular hydrostatic pressure, and enhances the reabsorption of sodium in the proximal tubules. Furthermore, angiotensin II stimulates the synthesis of aldosterone, which in turn increases the reabsorption of sodium in the distal tubule and increases the endothelin-1 in the kidney, a potent vasoconstrictor, proinflammatory, and profibrotic peptide. Angiotensin II also plays a role in other tissues, especially in the heart, where it promotes the process of remodeling and fibrosis.

Dysregulation of adenosine and arginine vasopressin (AVP) is also implicated in the neurohormonal pathogenesis of CRS. Adenosine is generated by enzymatic degradation of adenosine triphosphate. It is released when there is an increment of sodium levels in the distal tubule. After binding to A1 receptors, it causes vasoconstriction of afferent arterioles, reducing renal blood flow. Activation of A2 receptors stimulates renin secretion, increasing sodium reabsorption in the proximal tubule and decreasing the glomerular filtration. The use of drugs that block adenosine receptors can be useful in improving renal function. Unfortunately, clinical trials have failed to demonstrate the beneficial support of this therapy in reducing death and hospitalization, and improving heart and kidney function [31].

AVP is a peptide synthesized in the supraoptic and paraventricular nuclei of the hypothalamus that is stored in the posterior pituitary gland until released into the blood based on the serum osmolality. It increases the solute-free water reabsorption in the distal tubule of the kidney promoting aquaporin 2 (AQP2) movement from the intracellular compartment to the apical membrane. AQP2 is a water channel that drives water into the cell according to the osmotic gradient. In addition, AVP causes arteriole vasoconstriction, which increases vascular resistance. This mechanism leads to an increase in venous return to the heart, which worsens venous congestion. On the other hand, the vasoconstriction reduces kidney perfusion, resulting in GFR reduction. Several studies have evaluated the efficacy of tolvaptan, a selective, competitive AVP receptor antagonist, in CRS. The most important trial (EVEREST) recruited 4133 patients with ADHF, and found that early use of tolvaptan was linked to a lower mean body weight and ameliorated clinical symptomatology. Nevertheless, compared with placebo, tolvaptan effected no difference in long- term outcomes [32]. Based on the pathophysiology of the CRS, much attention was paid to AVP as a biomarker. However, the measured values of AVP are not always reliable [29]. Copeptin is the C-terminal portion of the AVP, and is easier, and more measurable and stable than AVP. Copeptin was shown to be correlated with CVD in patients with CKD [33].

### 2.4. Endothelial Dysfunction and Atherosclerosis

The neurohormonal dysregulation and the accumulation of the uremic toxins in CRS play an important role in the development of oxidative stress. The alteration of balance between oxidant and antioxidant agents results in an increased concentration of reactive oxygen species (ROS), leading to cellular damage and endothelial dysfunction.

Endothelial dysfunction is another important commonality between chronic kidney disease (CKD) and HF. Endothelial cells are responsible for the functional vascular response to hemodynamic and oxidative stress [34]. The anti-inflammatory, antioxidant action, and vascular tone regulation of the endothelium-derived relaxing factor (EDRF) are dysregulated in both HF and CKD independent of each other [35]. Endothelial dysfunction, on the other hand, is an early marker of atherosclerosis. Arteriosclerotic vascular disease (ASVD) plays a primary role in the pathogenesis of CRS. As is well known, ASVD (and consequent ischemic coronary artery disease) is both the main cause of HF and one of the main causes of ischemic renal failure [36]. The increased cardiovascular (CV) risk related to CKD is partly linked to a more intense and faster development of atherosclerosis, independent of the other risk factors [37].

### 2.5. Oxidative Stress and Inflammation

In CRS the increased oxidative stress is also related to ischemic injury and venous congestion, resulting in the reduction of fatty acid oxidation in favor of glycolysis in myocytes. These metabolism alterations lead to a reduction in ATP production, in turn leading to major susceptibility to hypoxemia, apoptosis, and cellular death. In addition, the reduced metabolism of fatty acid oxidation leads to an increase of free fatty acid and subsequent lipotoxicity.

Increased synthesis of pro-inflammatory mediators is known in CKD and HF to promote tissue damage, and lead to cell death and fibrosis. Cytokines, also induced through the extra-hemodynamic effects of angiotensin II, are fundamental in explaining the inflammatory mechanisms involved in CRS [38]. C-reactive protein (CRP), a non-specific inflammation protein, is frequently increased in CRS. It is associated with the activation of the complement system and tissue factor production. A study of acute decompensated heart failure syndromes prospectively assessed 4269 hospitalized AHF. In these patients, elevated CRP was independently associated with higher mortality (adjusted hazard ratio [HR], 1.68) [39].

As is well known, prolonged inflammation is often capable of inducing anemia in predisposed individuals. Anemia is a common characteristic of CKD and HF patients, with a prevalence between 5% and 55% [40], and represents an independent risk factor for mortality [41]. It contributes to CRS development in several ways: reduced oxygen delivery, reduced antioxidants synthesized from red blood cells, activation of sympathetic nervous system (SNS), RAAS, AVP because of tissue ischemia that leads to vasoconstriction, salt-water retention, and venous congestion. The role of erythropoiesis-stimulation agents (ESAs) in the treatment of CRS is controversial. A randomized double-blind controlled study demonstrated that improvement in hemoglobin levels with ESAs and oral iron over one year led to an increase in cardiac function compared with oral iron therapy alone [42]. On the contrary, a trial with a higher hemoglobin level (>13.5 gr/dl) as target found an association with a higher rate of adverse events and no improvement in the quality of life [43].

Finally, there is burgeoning interest in the adoption of parenteral iron to improve anemia in patients with congestive HF. Several studies [44] have reported that patients treated with intravenous iron develop symptomatic improvement and increased exercise capacity independent of hemoglobin level effects. These findings indicate that intravenous iron therapy can be a promising option for the treatment of CRS.

## 3. Biomarkers Connecting the Heart and Kidney

### 3.1. A New Look at an “Old” Biomarker: NT-proBNP

BNP and N-terminal (NT)-pro hormone BNP (NTproBNP) status are the gold standard biomarkers for HF, and one of the most used predictors of mortality and co-morbidities in these patients [45].

A strategy for stratifying CRS consists of monitoring the change in serum levels of NT-proBNP (N-terminal proBNP) and estimated mature BNP (em/BNP) in patients with acute decompensated HF. Takahama et al. [46] showed how the elevation of NT-proBNP/emBNP ratio precedes the worsening of renal function in patients with acute HF, and that this ratio was strongly associated with percentage decreases in eGFR. Mc Callum et al. [47] analyzed the data of 435 patients taken from the Ultrafiltration in Decompensated HF with CRS Study (CARRESS), and the Diuretic Optimization Strategies Evaluation (DOSE) trials. Their study aimed to assess the predictive value of kidney function reduction during decongestion in patients with acute decompensated HF.

The decline in NT-proBNP was associated with a lower risk of death and re-hospitalization (HR = 0.69 per halving, 95% CI 0.58, 0.83). The significant interaction (*p* = 0.002 unadjusted; *p* = 0.03 adjusted) between decrease in eGFR value and change in NT-proBNP confirmed that reduction of renal function during therapy for acute decompensated HF is linked to better outcomes if associated with a decline in NT-proBNP levels. The use of biomarkers of congestion, such as NT-proBNP, may be able to guide clinical decisions concerning the management of acute declines in eGFR. In fact, SCr, BNP, and NT-proBNP could provide guidance for patients with CRS. Rezk et al. [48] analyzed the data of 318 patients with CRS Type 5, linked to AL amyloidosis: an increase in NT-proBNP of >30% and a reduction in eGFR of ≥25% were independent predictors of death (HR 2.17, *p* = 0.009) and dialysis (HR 3.07, *p* = 0.002) at 6 months. At 12 months, a rise of NT-proBNP >30% was highly predictive of death (HR 3.67, *p* < 0.001) and dialysis (HR 2.85, *p* = 0.010). Truong et al. [49] assessed the cardiorenal involvement dosing NT-proBNP and cystatin C after CRT. Compared to subjects with low NT-proBNP and cystatin C, cardiorenal patients had a >9-fold increase risk of CRT unresponsiveness (odds ratio uncompensated 9.0; compensated 36.4; both *p* ≤ 0.004), and a >6-fold risk of Major Cardiac Risk Score (hazard ratio uncompensated 8.5; *p* = 0.005). The patients with elevated serum levels of both these biomarkers presented worse prognosis.

### 3.2. Biomarkers of CKD-Related Bone and Mineral Metabolism and CRS

Alterations in chronic kidney disease-related bone and mineral metabolism have emerged as novel risk factors for CV disease (CVD) mortality in patients with CKD and CRS [50].

Clinical studies have demonstrated that hyperphosphatemia is strongly linked with vascular calcification and CV mortality among dialysis patients [51]. Phosphate homeostasis is guaranteed by the correct function of the fibroblast growth factor-23 (FGF23) and its phosphaturic action. Elevated serum FGF23 levels and phosphate are independently related to a rise of all-cause and CV mortality in CKD patients [52].

FGF23 has not only been identified as a robust predictor of CVD outcomes in CKD patients, but recent studies have shown a connection between higher FGF23 levels and mortality, though patients had preserved renal function [53].

Milovanova et al. [54] analyzed the serum levels of FGF23 in 70 patients with different stages of CKD. Forty-nine hypertensive patients presented an immediate solid relationship between higher serum FGF-23 values and the increased posterior left ventricular wall (*r* = 0.552; *p* < 0.01) and, conversely, a strong one between the reduced serum Klotho levels and the ventricular wall thickness (*r* = −0.587; *p* < 0.01). Equivalent strong associations were found between higher serum levels of FGF-23 (*r* = 0.492; *p* < 0.01) and Klotho concentration (*r* = −0.537; *p* < 0.01) with peripheral vascular resistance index.

Scialla et al. [55] found in a cohort of 3860 CKD patients that elevated FGF23 was associated with congestive HF (CHF) more strongly than with atherosclerotic events (*p* = 0.02), and uniformly it was related to a greater risk of CHF (hazard ratio [HR], 1.45 per doubling [95% confidence interval (CI), 1.28 to 1.65]; HR for highest versus lowest quartile, 2.98 [95% CI, 1.97 to 4.52]), and atherosclerotic events (HR per doubling, 1.24 [95% CI, 1.09 to 1.40]; and HR for highest versus lowest quartile, 1.76 [95% CI, 1.20 to 2.59]). Their results suggest that FGF-23 might influence a CHF mechanism that mediates at least a part of excess CV risk due to CKD. 

At the kidney level, the interaction among FGF23, α-Klotho, and the fibroblast growth factor receptor (FGFR) induce phosphate excretion and a reduction of 1,25(OH)_2_ vitamin D synthesis that leads to a suppression of the intestinal phosphate reabsorption. The mechanisms of renal damage cause a decrease of α-Klotho, leading to FGF23 resistance and less efficient phospaturic action [56].

The principal control on FGF23 is carried out by 1,25(OH)_2_ vitamin D, which stimulates FGF23 creation through an endocrine feedback circuiting, modulating 1,25(OH)_2_ vitamin D production [57].

Notwithstanding the pleiotropic effects that vitamin D exerts to prevent different conditions leading to renal impairment [58,59,60] to modulate renin-angiotensin-aldosterone system and to reduce CV risk [61], a connection with this hormone has not been fully validated in CRS patients. Mann et al. [62] demonstrated that deficient serum levels of vitamin D are linked to a suppression of resting cardiac autonomic activity, while low 1,25-dihydroxy vitamin D levels correlated with unfavorable cardiac autonomic activity during an acute angiotensin II stressor challenge. Parker et al. [63], in their meta-analysis, examined the link between 25(OH) vitamin D levels and cardiometabolic disorders: a higher level of this hormone was linked to a 43% reduction in cardiometabolic dysregulation. The results of the study demonstrated that higher levels of vitamin D were associated with a considerable reduction of CVD, type 2 diabetes, and metabolic syndrome [64]. However, these considerations are speculative, the current literature presents methodological limitations, and it is still uncertain whether vitamin D low levels can have a predictive role in CVD and CRS outcomes.

### 3.3. Promising Biomarkers for Monitoring and Identifying Cardiorenal Syndrome

In the last few years new biomarkers have been increasingly investigated to find an easy-to-perform and highly predictive tool for assessing, monitoring, and guiding choices for CRS treatment.

A promising connection between cardiac geometry and serum hepcidin in CKD patients was found by Kim et al. [65]. On univariate analysis, both relative wall thickness and left ventricular mass index were associated with high serum hepcidin, where each 0.1 unit increase in relative wall thickness was associated with a 1.292-fold increase in the odds for high serum hepcidin *(p* < 0.001), while each 1 g/m^2.7^ increase in left ventricular mass index was associated with a 1.016-fold increase (*p* < 0.001). 

Recently, Nikorowitsch et al. [66] identified in the soluble urokinase-type plasminogen activator receptor (suPAR) a valuable biomarker for risk estimation in coronary artery disease. At Cox regression analyses, the hazard ratio related to CV death and/or myocardial infarction was 2.19 (*p* < 0.001) in the overall cohort and 2.56 in the acute coronary syndrome cohort (*p* < 0.001). The prognostic value of suPAR remained valid after adjusting for conventional CVD risk factors or conventional biomarkers such as C-reactive protein, NT-proBNP, and hs-cTnI a nd eGFR. In fact, a strong Spearman correlation coefficient was observed for eGFR, with a value of −0.27, (*p* < 0.001).

Galectin-3 is a recent biomarker belonging to the beta-galactosidase-binding lectin family, produced by activated macrophages and interacting with specific extracellular matrix proteins such as laminin, synexin, and integrins. The Ludwigshafen Risk and Cardiovascular Health (LURIC) study and German Diabetes Mellitus Dialysis (4D) study demonstrated that galectin-3 concentrations increase with progressive renal impairment [67]. Iacoviello et al. [68] evaluated the association between Gal-3 serum levels and the progression of renal dysfunction in 260 chronic HF patients: in these subjects Gal-3 was associated with a 1-year worsening of renal function on univariate analysis (odds ratio: 1.12; 95% CI: 1.06–1.18; *p* < 0.001).

Another promising biomarker proposed for CRS status assessment is the placental growth factor (PlGF), a member of the vascular endothelial growth factor family of cytokines; this molecule has a relevant role both in CRS and in CKD alone, predicting adverse events in the course of renal impairment. Nakada et al. [69] measured PlGF levels in a population of 408 patients with acute decompensated HF: Kaplan–Meier analysis revealed that the patients with the highest PlGF (quartile 4: ≥12.6 pg/mL) had poorer outcomes compared to the PlGF group (quartiles 1–3: <12.6 pg/mL) in terms of all-cause (hazard ratio [HR], 1.56; 95% confidence interval [CI], 1.13–2.14; *p* < 0.01) and CV death (HR, 1.68; 95% CI, 1.04–2.66; *p* < 0.05). 

An emerging biomarker of CRS is urine podocin/creatinine ratio (UP/Cr). Szczepankiewicz et al. [70] analyzed this ratio in a population of 50 dogs: they divided them into three groups: fifteen (control group), twenty-nine with degenerative mitral valve disease (heart group) and six with CKD (kidney group). Mean value ± standard deviation for UP/Cr was, respectively, 9.7 ± 4.8 × 10^−10^ for the control group, 49.0 ± 80.0 × 10^−10^ for the heart group, and 33.7 ± 18.0 × 10^−10^ for the kidney group. The UP/Cr in the heart and kidney groups was significantly higher than in the control group (*p* < 0.0001, sensitivity 0.83, specificity 0.20). 

Urinary cofilin-1 is another intriguing biomarker that acts as a modulator of epithelial-mesenchymal transition or de-differentiated renal tubular cells, which are essential for AKI and renal function recovery [71], and is related to severity of HF [72]. The group of Hsing-Yu Chen et al. [73] measured cofilin-1 in 44 patients: 13 patients were diagnosed with CRS, while the other 31 patients were classified into a non-CRS group; they predicted the occurrence of CRS and differentiated CRS patients from non-CRS patients, using the gold nanoparticle-based localized surface plasmon-coupled fluorescence biosensor with a significant accuracy (*p* = 0.031; overall accuracy 79.55%). 

Another panel of urinary proteins, already used for CV risk, could be used in CRS population: Martinez et al. [74] posed an interesting tool for assessing CV risk in a population of 81 patients (30–50 years old) divided into three groups: healthy patients, patients with CV risk factors, and patients who had previously suffered CV events. They identified 4309 proteins by mass spectrometry in urine samples, and from them selected six proteins used as molecular indicators associated with CV risk. Adrenodoxin (ADX), eosinophil cationic protein (ECP), fetuin B (FETUB), growth differentiation factor 15 (GDF15), guanine deaminase (GUAD), and neurogenic locus notch homolog protein 1 (NOTCH1) showed a positive correlation as urinary proteins linked to CV risk in all cases (Spearman correlation values: ADX *r* = 0.4889 *p* < 0.0001; ECP *r* = 0.6221 *p* < 0.0001; FETUB *r* = 0.4762 *p* < 0.0001; GDF15 *r* = 0.5570 *p* < 0.0001; GUAD *r* = 0.5549 *p* < 0.0001; and NOTCH1 *r* = 0.6493 *p* < 0.0001). This panel could be useful for foreseeing and preventing CRS features, even if dedicated study on these population should confirm its value. 

### 3.4. The Future Is Now: The Use of microRNAs for Cardiorenal Syndrome

The progressive affirmation of microRNAs (miRNAs) in clinical practice also involved CRS. MicroRNAs (miRNAs) are non-coding RNA molecules encoded in the human genome, 18 to 25 nucleotides in length, and responsible for post-transcriptional gene expression by binding to the 3’-untranslated region (UTR)/coding sequence (CDS) of mRNA of target genes. A number of studies have reported that microRNAs contribute either in a beneficial or negative way in mechanisms involving atherosclerosis and arterial remodeling, such as endothelial dysfunction, monocyte activation and migration in the arterial wall, and plaque constitution [75,76,77]. Several miRNAs have been reported in both acute and chronic state of primary organ failure in CRS (PMID: 34276211). A recent meta-analysis demonstrated that hsa-miR-423-5p can be used as a marker of HF, with a higher specificity if used in combination with BNP [78]. 

An miRNA that might play a decisive role in CVD management is the hsa-miR-21 that has been reported in all types of CRS and is associated with poor outcomes in most primary organ disfunctions [79]. It is known that miRNAs are involved in cardiac remodelling and regeneration processes through a balance of antifibrotic and profibrotic mechanisms. MiR-21 enhances EKR-MAPK activity which leads to fibroblast activation/proliferation causing renal and cardiac fibrosis [80]. Beyond the classic pathway of fibrosis modulation, miR-21 can further induce cardiac fibrosis through activation of collagen and a-SMA protein expression [81]. In addition, miR-21 would also participate indirectly in the process of fibrosis playing a role in the endothelial-mesenchymal transition (EndMT), indicating its multifunctional function in cardiac fibrosis [82]. In the kidney miR-21 contributes to the progression of acute and chronic kidney disease. Inflammation plays a key role in this process as it determines the activation of neutrophils that through the production of interleukins promotes the expression of miR-21 in renal fibroblasts. High renal miR-21 levels inhibit Notch2 expression which induces increased fibrosis markers such as collagen 1 and a-SMA. Furthermore miR-21 participates in renal fibrosis by targeting DDAH1 which is a key gene involved in the pathological process of ESRD [83]. MiR-21 can also be transported by microvesicles to other cell types to induce fibrosiss and it is upregulated in both B and T cells which induces strong inflammation. Interestingly, Wang et al. [84] documented in a population of elderly patients that serum hsa-miR-21 was an independent influencing factor for CRS (OR: 17.246; 95% CI: 3.334–89.218; *p* = 0.001). The AUC of hsa-miR-21 based on ROC curve was 0.749, with a sensitivity of 55.95% and a specificity of 84.93%. Furthermore, combining hsa-miR-21with cystatin C enhanced the AUC to 0.902, with a sensitivity of 88.1% and a specificity of 83.6% (*p* < 0.001). These data indicate the utility of hsa-miR-21 in identifying risk factors for CRS and better monitoring the status of these patients, especially if combined with cystatin C.

Other miRNAs were found during CRS but their role is not yet completely understood. A panel of five circulating miRNA has been successfully tested by Keller et al. [85] in the context of primary prevention of CVD. The population was represented by a group of low- to intermediate-patients presenting to their primary care physician and a group of a population-based sample excluding individuals with prevalent CVD.

They compared the hsa-miR-34a, -miR-223, -miR-378, -miR-499 and -miR-133 expression panel with the Framingham risk score (FRS), demonstrating a significant association with mortality given by a hazard ratio (HR) of 3.0 (95% (CI): 1.09–8.43; *p* = 0.034) and of 2.9 (95% CI: 1.32–6.33; *p* = 0.008) after adjusting for the FRS in a derivation cohort. 

A recent meta-analysis demonstrated that hsa-miR-423-5p can be used as a marker of HF, with a higher specificity if used in combination with BNP [78], left ventricular eject fraction, and BNP levels [86].

A recent in silico analysis aimed to identify crucial miRNAs and their associated target genes to pave the way for finding novel pathological mechanisms and biomarkers for CRS. Through this approach, the authors found that 5 miRNAs, namely, hsa-miR-122-5p, -miR-222-3p, -miR-21-5p, -miR-146a-5p, and -miR-29b-3p can be considered key regulators of CRS and correlate with it [87].

The next goal could be the creation of a multivariable panel of miRNAs combined with other conventional biomarkers to conceive accurate diagnostic and prognostic algorithms for CVD patients [88].

A summary of the biomarkers linked to CRS is represented in Table 1.

## 4. Conclusions

Previous valid papers aimed to enlighten the usefulness of classical CRS [90,91,92,93]. The purpose of our work was to underline the pathophysiological processes at the basis of CRS and how the most recent cardiorenal biomarkers can take place in the treatment strategy of this condition.

In this setting, the use of microRNA, the ratio between well-known kidney and heart dysfunction parameters, and novel panels of multiple biomarkers were analysed with their strength and weakness.

There is growing evidence regarding the validation and utility of recent serum and urinary biomarkers for acute and chronic CRS thanks to the harnessing of newer technologies or the development of dedicated protein panels. The use of high specificity and sensitivity tools with high prognostic values can help the clinician to overcome therapeutic limits and create new strategies for the treatment of complex clinical conditions [89,91].^.^

The use of accurate and reproducible markers can be helpful in various aspects of CRS treatment, including prevention of CVD events, monitoring patients even at the onset of the disease complications. These biomarkers can help clinicians choose more accurately the timing of a specific treatment and study adequate prevention strategies. Further studies will be fundamental in developing the best tools for improving the diagnosis, predicting adverse outcomes, and choosing the right treatment for CRS. 

## Figures and Tables

**Table 1:** List of biomarkers linked to CRS outcomes and their characteristics.

Biomarker	Description	References
Renal Biomarkers		
Serum creatinine (SCr), albuminuria, cystatin C (CysC), potassium, eGFR	Classical biomarkers for the assessment of kidney function. Evidence for several indicators in CRS, especially when combined, has increased in recent years. For example, reduced albuminuria and potassemia indicate early renal repair processes after resynchronization therapy in CRS2.	Gala-Błądzińska et al. [16]
TIMP2•IGFBP7	Urinary tissue inhibitor of metalloproteinase-2 • Insulin-like growth factor–binding protein 7 (TIMP2•IGFBP7). This is a combined marker of acute kidney damage. It can predict the onset of moderate to severe AKI, and is elevated in CRS patients.	Gunnerson et al. [22]
Urinary angiotensinogen	A marker of acute kidney damage and a strong predictor of worsening of AKI, with death in acute decompensated heart failure. The urinary angiotensinogen measured at the time of CRS diagnosis shows improved risk stratification.	Chen et al. [13]
NAG	Urinary enzyme N-acetyl-β-d-glucosaminidase (NAG). Measured at the time of CRS diagnosis, it shows improved risk stratification in determining which patients will experience adverse outcomes.	Chen et al. [13]
KIM-1	Urinary kidney injury molecule 1 (KIM-1). Measured at the time of CRS diagnosis, it shows improved risk stratification in determining which patients will experience adverse outcomes.	Chen et al. [13]
NGAL	Urinary and serum neutrophil gelatinase associated lipocalin (NGAL). It is primarily used (especially its urinary determination) as early marker of renal damage.	Chen et al. [13] Cernaro et al. [15]
L-FABP	Liver fatty acid-binding protein (L-FABP). This is a marker of acute kidney damage in acute decompensated heart failure.	Hishikari et al. [23]
b2M and TIMP1	β2-microglobulin (b2M) and tissue inhibitor of metalloproteinases 1 (TIMP1). Their values have been demonstrated to be potentially linked to severity of CRS.	Vianello et al. [21]
UP/Cr	Urine podocin/creatinine ratio (UP/Cr) is an emerging biomarker of CRS. In a study conducted in dogs, the UP/Cr in the CVD and CKD groups was significantly higher than in the control group.	Szczepankiewicz et al. [89]
Cardiac Biomarkers		
hs-cTnI	High sensitivity troponin I (hs-cTnI). Marker of myocardial injury, which increases with declining eGFR. Sustained elevation is associated with a higher mortality risk.	Chen et al. [13]
Galectin-3	A marker of cardiac fibrosis, stress and remodeling. It is associated with the progression of renal dysfunction in patients with heart disease.	Iacoviello et al. [90]
Neurohormones and hormones		
BNP; NT-proBNP; emBNP	B-type natriuretic peptide (BNP) and N-terminal proBNP (NT-proBNP) are the gold standard biomarkers for heart failure. The elevation of the NT-proBNP/estimated mature BNP (emBNP) ratio precedes the worsening of renal function in patients with acute HF, and has been strongly associated with decreases in eGRF.	Kociol et al. [45]
AVP and copeptin	Copeptin is the C-terminal part of the arginine vasopressin (AVP), and is an easily measurable and stable molecule in plasma. Copeptin could be correlated with CVD in patients with CKD or ERSD.	Yalta et al. [33]
Oxidative stress and inflammatory biomarkers		
XOA	The increased xanthine oxidoreductase activity (XOA), the elevated CysC value and reduced eGFR are significantly related to risk of acute CRS.	Tasić et al. [17]
IL-18	Interleukin 18 (IL-18) is a cytokin used as a marker in acute kidney damage. Measured at the time of CRS diagnosis, it shows improved risk stratification in determining which patients will experience adverse outcomes.	Chen et al. [13]
Mineral and bone disorder biomarkers		
FGF-23	Fibroblast growth factor 23 (FGF-23) is involved in phosphate homeostasis. It could be used as marker of renal and heart failure.	Jean et al. [71] Scialla et al. [74]
Vitamin D	Vitamin D has pleiotropic effects and, as a marker, has a significant impact on the cardiovascular, nervous, endocrine and immune systems. However, its role in CRS patients is not clear.	Mann et al. [81] Binanay et al. [10]
MicroRNAs		
MiR-21; miR-122-5p, miR-222-3p, miR-21-5p, miR-146a-5p, miR-29b-3p	MicroRNAs (miR) in clinical practice also involves CRS. MiR-122-5p, miR-222-3p, miR-21-5p, miR-146a-5p, and miR-29b-3p, correlated with CRS. MiR-21 has been shown to be an independent influencing factor for CRS.	Yan et al. [78] Keller et al. [85] Wang et al. [84]
Other biomarkers		
MPV	Mean platelet volume (MPV). This might be a useful predictor of acute CRS prognosis.	Li et al. [67]
Hepcidin	There is a link between elevated serum hepcidin (high in anemia of inflammation that is hyporesponsive to erythropoiesis-stimulating agents) and cardiac geometry (relative wall thickness and left ventricular mass index). It is correlated with prognosis in patients with heart failure.	Kim et al. [88]
suPAR	Soluble urokinase-type plasminogen activator receptor (suPAR) might represent a valuable biomarker for risk estimation in coronary artery disease and HF. Its prognostic value remains valid also after adjusting for eGFR.	Nikorowitsch et al. [91]
PlGF	Placental growth factor (PIGF) is a key molecule in CRS and a predictor of adverse events in chronic kidney disease patients.	Nakada et al. [92]
Urinary cofilin-1	Acts as a modulator of epithelial-mesenchymal transition or de-differentiated renal tubular cells, which are essential for AKI and renal function recovery, and is related to severity of HF. It is a highly potential biomarker for predicting CRS among coronary care unit patients.	Rastaldi et al. [91] Huntley et al. [72] Chen et al. [73]
ADX, ECP, FETUB, GDF15, GUAD, NOTCH1	Urinary adrenodoxin (ADX), eosinophil cationic protein (ECP), fetuin B (FETUB), growth differentiation factor 15 (GDF15), guanine deaminase (GUAD) and neurogenic locus notch homolog protein 1 (NOTCH1) are a panel of urinary proteins, already used for CV risk, that might be useful in foreseeing and preventing CRS features.	Martinez et al. [74]

Seric and urinary biomarkers that can be potentially used for CRS outcomes. These indicators have the additional potential to identify individuals susceptible to disease in order to study the best strategies for CRS treatment. These biomarkers can be used individually and in combination. Abbreviations: AKI: acute kidney injury. CRS: cardiorenal syndrome. CV risk: cardiovascular risk. CVD: cardiovascular disease. CKD: chronic kidney disease. eGFR: estimated glomerular filtration rate. ERSD: end-stage renal disease. HF: heart failure.

## Data Availability

This study did not report any new data.
